# Pipeline analysis of a vaccine candidate portfolio for diseases of poverty using the Portfolio-To-Impact modelling tool

**DOI:** 10.12688/f1000research.19810.2

**Published:** 2020-02-04

**Authors:** Alexander Gunn, Shashika Bandara, Gavin Yamey, Flavia D´Alessio, Hilde Depraetere, Sophie Houard, Nicola K Viebig, Stefan Jungbluth

**Affiliations:** 1 Center for Policy Impact in Global Health, Duke Global Health Institute, Duke University, Durham, USA; 2 European Vaccine Initiative (EVI), Heidelberg, Germany

**Keywords:** European Vaccine Initiative, vaccines, diseases of poverty, emerging infectious diseases, portfolio, P2I

## Abstract

**Background:** The Portfolio-To-Impact (P2I) P2I model is a recently developed product portfolio tool that enables users to estimate the funding needs to move a portfolio of candidate health products, such as vaccines and drugs, along the product development path from late stage preclinical to phase III clinical trials, as well as potential product launches over time. In this study we describe the use of this tool for analysing the vaccine portfolio of the European Vaccine Initiative (EVI). This portfolio includes vaccine candidates for various diseases of poverty and emerging infectious diseases at different stages of development.

**Methods:** Portfolio analyses were conducted using the existing assumptions integrated in the P2I tool, as well as modified assumptions for costs, cycle times, and probabilities of success based on EVI’s own internal data related to vaccine development.

**Results:** According to the P2I tool, the total estimated cost to move the 18 candidates currently in the EVI portfolio along the pipeline to launch would be about US $470 million, and there would be 0.69 expected launches across all six diseases in EVI’s portfolio combined during the period 2019-2031. Running of the model using EVI-internal parameters resulted in a significant increase in the expected product launches.

**Conclusions:** Not all the assumptions underlying the P2I tool could be tested in our study due to limited amount of data available. Nevertheless, we expect that the accelerated clinical testing of vaccines (and drugs) based on the use of controlled human infection models that are increasingly available, as well as the accelerated approval by regulatory authorities that exists for example for serious conditions, will speed up product development and result in significant cost reduction. Project findings as well as potential future modifications of the P2I tool are discussed with the aim to improve the underlying methodology of the P2I model.

## Introduction

The Special Programme for Research and Training in Tropical Diseases (TDR) recently developed a new modelling tool, called Portfolio-to-Impact (P2I), that allows users to model the impact of different research portfolios ^1^. The P2I tool can be used to estimate the costs of moving a portfolio of candidate products for poverty-related and neglected diseases (PRNDs) through the pipeline and the likely launches that would result. It can also help to identify potential funding bottlenecks and operational challenges. The modelling tool, which is deterministic, uses Excel based software to estimate the “minimum funding needs to accelerate health product development from late stage preclinical study to phase III clinical trials” and to “visualize potential product launches over time ^1^.”

As a financial forecasting tool that estimates the funding needs for pharmaceutical product development, the tool and its outputs are of value to funders of product development, product development partnerships, and other stakeholders involved in research and development (R&D) policy. Terry and colleagues, who developed the P2I model, note that “its real utility lies in its predictive value for modelling the impact of different funding strategies at the portfolio level” ^1^.

To the best of our knowledge, the first published use of the tool was by Young and colleagues ^2^. These researchers first conducted a pipeline portfolio review to identify current candidates in the pipeline for 35 PRNDs. They then used the P2I tool to estimate (a) the costs to move these candidates through the pipeline, (b) the likely launches, and (c) the highly needed products that would still be “missing” at the end. As of August 31, 2017, they found 685 PRND product candidates, of which 538 candidates met inclusion criteria for input into the model. Their modelling estimated that it would cost about US$ 16.3 billion (range $13.4–19.8B) to move these candidates along the pipeline, resulting in about 128 (89–160) expected product launches. The study found that “there would be few launches of complex new chemical entities; launches of highly efficacious HIV, tuberculosis, or malaria vaccines would be unlikely ^2^.”

The European Vaccine Initiative (EVI), established in 1998 as the European Malaria Vaccine Initiative (EMVI), is a not-for-profit organization that supports the development of effective, affordable and accessible vaccines against diseases of poverty and emerging infectious diseases. To achieve this goal, EVI supports translational vaccine research and development (R&D) spanning from preclinical development through to the establishment of a clinical proof of concept. To date, EVI has supported the development of about 40 vaccine candidates through to early- and mid-stages of clinical development. Initially focussing only on malaria vaccines, in 2009 in the context of a strategic revision EVI broadened its scope and since has built a vaccine portfolio that addresses critical challenges and opportunities in vaccine R&D for a variety of diseases of poverty and emerging infectious diseases.

Currently EVI´s vaccine portfolio comprises around 20 vaccine candidates at different stages of development between late preclinical and mid-stage clinical development. In order to estimate future financing needs required for delivering the EVI portfolio and the potential public health impact of product launches, we conducted an analysis of EVI´s vaccine candidate portfolio using the P2I tool.

Together with similar pipeline portfolio reviews using the P2I tool that are currently being conducted by other product development partnerships (PDPs), the results will inform product developers as well as funders and policy makers regarding future funding needs. The results may also guide future investment priorities to maximise the chances of developing products for diseases that are missing urgently needed health products.

We used the P2I tool because, to the best of our knowledge, it is the first publicly available comprehensive portfolio model that includes data on cost, success rate, and cycle time per phase for various product types based on data from a very large number of previous product development candidates (over 25,000) ^1^. The P2I tool is thus complementary to other available tools that can help guide prioritization in vaccine development, such as the multi-stakeholder Vaccine Innovation Prioritization Strategy ^1^ and Total Systems Effectiveness Framework ^2^.

## Methods

**Figure 1.**
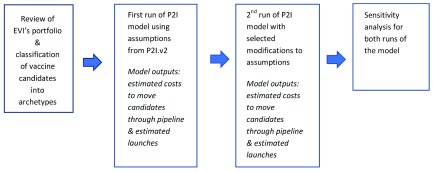
Four key phases in the analysis.

In this section, we begin by summarizing how the Microsoft Excel-based P2I tool was developed, which phases of product development are included in the tool, and which costs are excluded. After this summary, we then describe the four key steps in our analysis of EVI’s vaccine portfolio, which are summarized in Figure 1.

### A summary of how the P2I tool was developed, and which phases and costs are included

**Figure 2.**
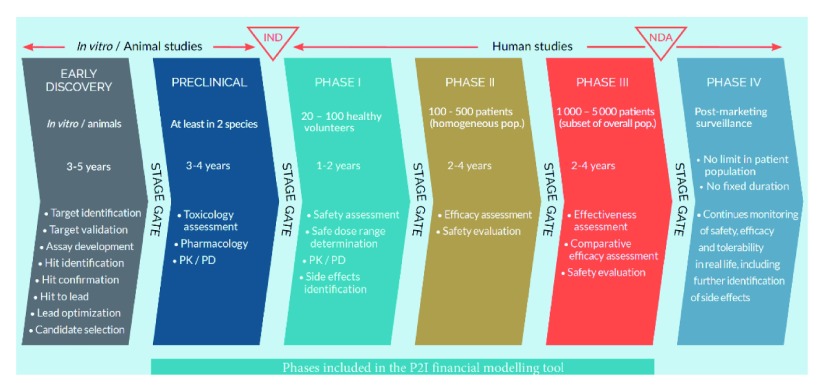
Development phases included in the P2I model. ND: investigational new drug application; NDA: new drug application; m: million; PK: pharmacokinetics (absorption, distribution, metabolism, excretion); PD: pharmacodynamics; pop.: population; P2I: Portfolio-to-Impact (adapted from Health Product Research and Development Fund: A Proposal for Financing and Operation ^3^).

A detailed research paper describing the development of the first version of the P2I tool itself (P2I version 1, or P2I v1) has been previously published ^1^. The development of a second version of the P2I tool, called P2I version 2 (P2I v.2), has also been previously described ^2^. As summarized below, the model is based on assumptions for costs per phase, attrition rates (probability of success) per phase, and cycle times per phase for four development phases (preclinical to phase III, see Figure 2) for a number of different kinds of medical products, called archetypes.

The P2I v.1 model has 11 archetypes: simple or complex vaccines; simple, innovative, or complex new chemical entities (NCEs); simple or complex repurposed drugs; simple or complex diagnostics; simple or complex biologics; and two diagnostic archetypes, assay development or simple technical platform development. Descriptions and examples of each are described elsewhere ^1^. For each of these 11 different archetypes, the model has in-built assumptions on costs, attrition rates, and cycle time per phase.

One valuable feature of the model is that it is highly adaptable, users can input additional archetypes into the Excel tool. In the P2I v.2 model, additional archetypes include “unprecedented vaccines” (for candidate vaccines for HIV, TB, and malaria to, which are “considered as unprecedented as current platforms have not led to suitable vaccines” ^2^) and vector control products.

As described elsewhere ^1^, assumptions on development costs at each phase of product development for the 11 archetypes included in the P2I.v1 model were initially based on an analysis of clinical trial costs from Parexel’s R&D cost sourcebook. The assumptions on attrition rates and cycle times at each phase were initially based on the historical attrition rates and cycle times of more than 25,000 development candidates. All of these assumptions were further refined and validated based on (i) academic literature, (ii) industry publications and databases, and (iii) 133 stakeholder interviews with a wide variety of product development partnerships (PDPs), pharmaceutical companies, and major funders of global health R&D. As described elsewhere ^2^, additional sources of assumptions for the new archetypes in P2I.v2 were the McKinsey Risk-Adjusted Portfolio (RAP) Model and clinical trial data shared by the Bill & Melinda Gates Foundation.

As described below, the three archetypes of relevance for our analysis of the EVI portfolio were simple, complex, and unprecedented vaccines (reference 2 describes these three different archetypes in more detail). After classifying each EVI candidate into its archetype and phase, we then ran the model. There are two main model outputs. The first is “launches”; in this paper, the term launch refers to a candidate making it through phase III and thus being ready for the next steps, e.g. the regulatory and manufacturing steps. The second is the total costs to move all candidates through the pipeline from their current phase from now to 2031 (the model also gives a breakdown of these total costs into annual costs by year, from 2019 to 2031).

The model includes only advanced preclinical to phase III, and thus the cost estimates are an under-estimate of the full costs of product development. In particular, the model excludes all costs related to basic research through lead optimization; chemistry, manufacturing, and controls; good manufacturing practice; manufacturing build up and scale-up costs; regulatory or registration fees (post-phase III); and all post-market commitments (e.g., phase IV pharmacovigilance studies).

### Step 1: review of EVI’s portfolio and classification of vaccine candidates into archetypes

The first step was to review, organize, and classify the vaccine candidates to allow them to be entered into the P2I model. To enter candidates into the model, we needed to include (a) the target disease, (b) the current phase of development (the model assumes that the candidate is at the start of that phase), and (c) the archetype. Table 1 summarizes the vaccine candidates included in the analysis. Since all product archetypes were vaccines, we used the archetype classification from the P2I v.2 model ( Table 2), in which candidates are classified as simple, complex, or unprecedented.

**Table 1.** List of vaccine candidates, disease target, product archetype, and current development phase for EVI’s portfolio.

Candidate name	Disease target	Vaccine archetype	Development phase
*Malaria 1*	malaria	unprecedented	phase II
*Malaria 2*	malaria	unprecedented	phase I
*Malaria 3*	malaria	unprecedented	phase I
*Malaria 4*	malaria	unprecedented	phase II
*Malaria 5*	malaria	unprecedented	phase II
*Malaria 6*	malaria	unprecedented	preclinical
*Malaria 7*	malaria	unprecedented	preclinical
*Malaria 8*	placental malaria	unprecedented	phase I
*Malaria 9*	placental malaria	unprecedented	phase I
*Malaria 10*	malaria	unprecedented	phase I
*Malaria 11*	placental malaria	unprecedented	phase I
*Malaria 12*	placental malaria	unprecedented	phase I
*Malaria 13*	placental malaria	unprecedented	phase I
*Zika 1*	Zika	simple	phase I
*Nipah 1*	Nipah	simple	phase I
*Diarrheal disease 1*	shigellosis, ETEC *	complex	phase I
*Leishmaniasis 1*	leishmaniasis	unprecedented	preclinical
*Leishmaniasis 1*	leishmaniasis	unprecedented	phase II

Abbreviations: *ETEC: enterotoxigenic *E. coli*

**Table 2.** Classification of vaccine candidates into archetypes (based on references 1 and 2; published under CC-BY 4.0).

Archetype	Original description from P2I v.1	Examples	Additional description from P2I v.2
Simple vaccine	Platform has been used to develop other vaccines	Hep A, Hep B, polio, killed or live attenuated vaccines	Any vaccine platform that has been extensively researched and approved for use in the past. Pathogen has readily-identifiable vaccine targets that lack complexity. Conferral of immunity against disease- causing microorganism is expected as natural immunity to the pathogen is protective. Platform is likely to elicit robust protective response.
Complex vaccine	Requires completely novel approach; no platform; no existing research	Pneumococcal conjugate vaccine, meningitis B, DNA or mRNA vaccines	Any vaccine platform that requires a novel approach that has not been successfully approved for use in the past. Conferral of immunity against disease-causing microorganism is difficult to induce and maintain and natural immunity is not protective against reinfection. Platform may elicit incomplete/insufficient immunity and require boosting over time.
Unprecedented vaccine	Was not included as an archetype in P2I v.1	HIV, TB, malaria vaccines	All vaccine candidates for HIV, TB, and malaria are classified as “unprecedented” due to much higher attrition rates at phases II and III than other complex vaccines

### Step 2: modelling of costs to move candidates through pipeline and likely launches, using P2I v.2 model with its existing assumptions

Once EVI’s portfolio of candidates was classified into archetypes (see Table 1), we then entered them into the P2I v.2 model and ran the model. In this first run of the model, we used exactly the same assumptions on cycle time, cost, and attrition rate per phase as in the P2I.v2 model ^2^. These assumptions are shown in Table 3. The assumptions were derived from three sources: the P2I model (shown in orange in Table 3), the McKinsey Risk-Adjusted Portfolio (RAP) Model (shown in yellow), and the Bill & Melinda Gates Foundation (shown in blue) ^2^. The model outputs were the estimated costs to move the current candidates through the pipeline and the estimated number of launches.

**Table 3.** Assumptions on costs, cycle times, and probabilities of success per phase for simple, complex, and unprecedented vaccines from the P2I v.2 model (table adapted from reference 2 under a CC-By 4.0 license).

Archetype	Cost per phase ($, millions)				Length of phase (years)				Probability of success (%)			
	Preclinical	Phase 1	Phase 2	Phase 3	Preclinical	Phase 1	Phase 2	Phase 3	Preclinical	Phase 1	Phase 2	Phase 3
Simple vaccine	6.7	2.2	13.2	201.0	3.4	1.6	2.2	2.3	41.0	68.0	46.0	71.0
Complex vaccine	16.6	2.5	13.9	223.0	3.3	2.0	3.7	3.5	41.0	50.0	22.0	64.0
Unprecedented vaccine	16.6	2.5	13.9	223.0	3.3	2.0	3.7	3.5	41.0	50.0	5.0	40.0

**Table:** 

***Source of data for assumptions:***		
P2I model assumptions	McKinsey RAP	Bill & Melinda Gates Foundation

### Step 3: modelling of costs to move candidates through pipeline and likely launches, using the P2I v.2 model with modified assumptions

As a third step, we did a second run of the model after making selected modifications to some of the assumptions used in P2I v.2.

EVI has collected data on its own parameters for cycle times, attrition rates, and costs per phase, which are shown in Table 4. We wanted to assess what effect the use of EVI’s parameters would have upon the outputs of the P2I model, but we recognized that many of these parameters were based on only two or three data points and were thus unreliable. For the second run of the model, we therefore made a pragmatic decision to use only those EVI parameters that were based on 10 data points, i.e. EVI’s parameters on success rate in phase 1 and the duration of phase 1. Thus, in the second run of the model, we made two modifications:

- Instead of using a success rate of 50% for phase 1 for unprecedented vaccines (the assumption in P2I v.2, as shown in Table 3), we used EVI’s success rate of 70% (see Table 4).
- Instead of using a phase length of 2 years for phase 1 for unprecedented vaccines (the assumption in P2I v.2, as shown in Table 3), we used EVI’s phase length of 1.45 years or 17.4 months (see Table 4).

**Table 4.** EVI’s own data on costs, cycle times, and probabilities of success per phase (see Appendix 1 for additional details).

Stage	n=	Average value	Vaccine archetypes
Preclinical phase duration (months)	2	36	all unprecedented
Preclinical phase cost EUR	3	EUR 2,483,333	1 simple, 2 unprecedented
Technical success phase I	10	100%	all unprecedented
Phase transition success phase I	10	70%	all unprecedented
Duration phase I (months)	10	17.4	all unprecedented
Phase I cost EUR	3	EUR 1,500,000	all unprecedented
Technical success phase II	2	100%	all unprecedented
Phase transition success phase II	2	100%	all unprecedented
Duration phase II (months)	2	22.5	all unprecedented

For the EVI parameters that were included in the second run of the model, we used the following definitions:

- **Success rate:** EVI uses two different measures: (i) *technical success*: a clinical trial is considered successful if it concluded without being terminated prematurely for whatever reason, and (ii) *phase transition success:* a clinical trial is considered successful if after completion of the trial a decision is made to move to a subsequent clinical trial (even if no funding is available to do so; successful in this sense means also if you conduct, for example, another phase I clinical trial of the same antigen with a different formulation, dose, or age group).
- **Clinical trial duration**: EVI excludes clinical trial preparation, including dossier preparation and waiting for approval, in the duration. The duration is the period between the start and completion times. The start time is the time point when the clinical study opened for recruitment of participants, or the actual date on which the first participant was enrolled. The completion time is the time point when the final participant was examined or received an intervention for the purposes of final collection of data for the primary outcome.

### Step 4: sensitivity analysis

As a final step, we conducted a sensitivity analysis, using an approach developed by Mestre-Ferrandiz *et al*. at the United Kingdom Office of Health Economics ^3^. We examined the impact of changing all probabilities of success per phase to 10% higher and 10% lower, and all costs per phase to 10% higher and lower. We also examined the impact of all possible combinations of these changes (e.g., 10% higher probability of success per phase *and* a 10% higher cost per phase, 10% higher probability of success per phase *and* a 10% lower cost per phase, etc.). We conducted this sensitivity analysis for both runs of the model.

## Results

### (i) Review of EVI’s portfolio

A review of EVI’s portfolio identified a total of 18 vaccine candidates under development, which were entered into the P2I v.2 model ( Table 1). There were 8 candidates for malaria, 5 for placental malaria, 2 for leishmaniasis, and one each for shigellosis, Nipah virus, and Zika virus. Three candidates were in the pre-clinical phase, 11 in phase I, and 4 in phase II. There were no candidates in phase III. Among the 18 candidates, 15 were classified as unprecedented vaccines, 2 as simple vaccines, and one as a complex vaccine.

As described in the Methods section above, we first ran the model using the assumptions included in the P2I v.2 model. The results of this first run are described in section (ii) below. We then re-ran the model with some modifications to the assumptions that were made by EVI, based on EVI’s own historical data; section (iii) describes the results of this second run. Finally, we conducted a sensitivity analysis around the results of both runs of the model; the results of these sensitivity analyses are in section (iv).

### (ii) First run of the P2I financing modelling tool, using assumptions from P2I v.2

***Estimated costs to move candidates through the pipeline.*** The total estimated cost to move the 18 candidates for six diseases along the pipeline to launch would be about US $470 million ( Table 5). Just over one third (35%) of the expected costs would be incurred by development of the 8 vaccine candidates for malaria. Development of candidates for placental malaria, Nipah virus, and Zika virus would each account for about 16% of the total costs. The remaining costs would be for development of candidates for leishmaniasis (10% of costs) and shigellosis (7% of costs).

**Table 5.** Cost and launch probability per disease based on P2I v.2 model projections.

*Disease*	Cost (US$, millions)	Expected launches (probability of launch)
*Malaria*	165.62	0.098
*Leishmaniasis*	47.77	0.02
*Shigellosis, ETEC*	33.96	0.07
*Nipah*	73.96	0.22
*Zika*	73.96	0.22
*Placental malaria*	75.08	0.05
*Total*	470.35	0.69

***Expected launches (expressed as launch probabilities).*** Overall, for all 18 candidates under development, the P2I model estimates that there would be 0.69 expected launches across all six diseases combined, as shown in Table 5 (we have left all results unrounded). Table 5 summarizes the expected launches by disease based on the current candidates for six diseases in EVI’s portfolio (the “expected launches” throughout this paper are expressed as launch probabilities, where 1.0 is 100% probability of a launch). Table 6 summarize the estimated launches for all six diseases combined (as launch probabilities) by year from 2019–2031 alongside cost estimates by year to move candidates for all six diseases through the pipeline from their current phase.

**Table 6.** Cost and annual launch probability by year.

*Year*	Cost (US$, millions)	Launch probability (1 = 100% probability of a launch)
*2019*	44.11794	0
*2020*	90.39988	0
*2021*	145.5832	0
*2022*	199.9804	0
*2023*	285.8381	0
*2024*	373.1006	0
*2025*	413.073	0.44
*2026*	437.6135	0.52
*2027*	459.6735	0.52
*2028*	465.4747	0.67
*2029*	467.4339	0.67
*2030*	469.3932	0.67
*2031*	470.3567	0.69

### (iii) Second run of the P2I financing modelling tool, with modifications of selected assumptions

The re-run (second run) of the model using the modified assumptions increased the projected portfolio costs by US $46 million, bringing the total cost estimate to US$ 517 million to move all 18 candidates through the pipeline. The changes in estimated cost are driven by the increase in expected costs for the unprecedented vaccine candidates, i.e., the 8 malaria vaccine candidates, 5 placental malaria vaccine candidates and 2 leishmaniasis vaccine candidates. The remaining candidates for Nipah virus, Zika virus, and shigellosis were not affected by the change in parameters.

With regards to the expected number of launches, in the re-run of the model, the launch probability increased for malaria, placental malaria, and leishmaniasis vaccines:

- For malaria, the estimate of expected launches increased from 0.098 to 0.11
- For placental malaria, the estimate of expected launches increased from 0.05 to 0.07
- For leishmaniasis, the estimate of expected launches increased from 0.024 to 0.03.

Table 7 provides a comparative summary of the costs and launch probabilities for each run of the model.

**Table 7.** Comparison of model outputs based on original assumptions and EVI assumptions.

First Run of Model (Assumptions From P2I v.2)				Second Run of Model (Selected Assumptions Modified by EVI)	
Disease	Archetype	Total Cost (US$, millions)	Total Expected Launches (Launch Probabilities, where 1 = 100% probability)	Total Cost (US$, millions)	Total Expected Launches (Launch Probabilities, where 1 = 100% probability)
**Malaria**	Unprecedented	165.62	0.098	184.75	0.11
**Placental malaria**	Unprecedented	75.08	0.05	100.11	0.07
**Leishmaniasis**	Unprecedented	47.77	0.02	49.83	0.03
**Shigellosis, ETEC**	Complex	33.96	0.07	33.96	0.07
**Nipah**	Simple	73.96	0.22	73.96	0.22
**Zika**	Simple	73.96	0.22	73.96	0.22
**Total**		470.35	0.69	516.57	0.72

### (iv) Sensitivity analyses for both runs of the model

We conducted a sensitivity analysis for the first and second runs of the model.

***Sensitivity analysis for first run of the model.*** In the sensitivity analysis for the first run of the model (which used assumptions from P2I v.2), we found that the total estimated costs to move all candidates in EVI’s portfolio for all six diseases through the pipeline from their current phase ranged from US$ 417.08 million to US$ 528.11 million ( Table 8). The combined launch probability for launching candidates across all six disease types ranged from 0.51 to 0.91.

**Table 8.** Results of the sensitivity analysis for first run of the model.

Parameters	Percentage change from baseline	Effect on estimated cost of development		Effect on estimated number of product launches	
		Cost (US $, millions)	Delta (%)	Number of Launches (Launch Probabilities, where 1 = 100% probability)	Delta (%)
Baseline		470.35	-	0.69	-
Probability of success	Low (-10%)	417.08	-11.33	0.51	-26.1
	High (+10%)	528.11	12.28	0.91	31.9
Average cost per phase	Low (-10%)	464.91	-1.16	-	-
	High (+10%)	517.39	10.00	-	-
Probability of success, and average cost per phase	Low (-10% for both parameters)	410.7	-12.7	0.51	-26.1
	Intermediate 1 (Cost+10%, Probability of success -10%)	501.9	6.7	0.51	-26.1
	Intermediate 2 (Cost-10%, Probability of success +10%)	458.8	-2.5	0.91	31.9
	High (+10% for both parameters)	580.9	23.51	0.91	31.9

***Sensitivity analysis for second run of the model.*** In the second sensitivity analysis for the second run of the model (which used modified assumptions), we found that the total estimated cost to move all candidates in EVI’s portfolio for all six diseases through the pipeline from their current phase ranged from US$ 482.48 million to US$ 581.9 million ( Table 9). The combined launch probability for launching candidates across all six disease types ranged from 0.53 to 0.96.

**Table 9.** Results of the sensitivity analysis for second run of model.

Parameters	Percentage change from baseline	Effect on estimated cost of development		Effect on estimated number of product launches	
		Cost (US $, millions)	Delta (%)	Number of Launches (Launch Probabilities, where 1 = 100% probability)	Delta (%)
**Baseline**		516.57	-	0.72	-
**Probability of success**	Low (-10%)	482.48	-6.60	0.53	-26.4
	High (+10%)	581.90	12.65	0.96	33.3
**Average cost per phase**	Low (-10%)	464.91	-10.00	-	-
	High (+10%)	568.22	10.00	-	-
**Probability of success, and** **average cost per phase**	Low (-10% for both parameters)	410.7	-20.5	0.53	-26.4
	Intermediate 1 (Cost+10%, Probability of success -10%)	501.9	-2.8	0.53	-26.4
	Intermediate 2 (Cost-10%, Probability of success +10%)	523.7	1.4	0.96	33.3
	High (+10% for both parameters)	640.1	23.91	0.96	33.3

## Conclusions and discussion

The mission of EVI is to accelerate the development of vaccines for diseases of poverty and emerging infectious diseases. Compared to other PDPs with a narrower focus, for example on a single disease, EVI has a broader scope and consequently a more heterogeneous portfolio, currently comprising 18 active vaccine candidates covering five different diseases/pathogens (malaria, including placental malaria, leishmaniasis, shigella/ETEC, Nipah and Zika viruses). An analysis of EVI´s current vaccine portfolio, providing estimations for future vaccine development costs and expected product launches, was considered important to inform future decision-making and priority setting at EVI, as well as to provide valuable information to global health funders and policy makers. At EVI, decision regarding which vaccine candidates to include into the organisation’s portfolio, and which ones to move forward, are based on a rigorous selection of candidates made by the EVI Board, based on input from an independent scientific advisory committee. For selecting vaccine candidates and advancing their development, EVI employs a portfolio management approach that has defined gating criteria (Go/No-Go criteria), ensuring that only the best leads are fed in and candidates that do not meet the criteria set are weeded out early on, thereby balancing the number of projects supported with available resources ^4^. Results delivered by the portfolio analysis using the P2I tool, in particular the total estimated costs and expected success rates, therefore provide valuable information that informs this selection and decision-making process.

Overall, using the pre-defined assumptions established in the P2I tool, our modelling resulted in a total estimated cost of about US $470 million for moving all 18 candidates included in the analysis along the pipeline until launch. Of this total amount, just over one third (35%) of the expected costs would be incurred by the development of the eight vaccine candidates for malaria (excluding those for placental malaria). Development of the candidates for placental malaria, Nipah virus, and Zika virus would account for about 16% each of the total costs. The remaining financing would be required for the development of candidates for leishmaniasis and shigellosis (10% and 7% of total costs, respectively).

The re-run (second run) of the P2I model using modified assumptions for phase costs and length based on EVI’s internal data increased the projected portfolio costs by US $46 million up to a total cost of US$ 517 million for all 18 candidates. The main driver of this change in the estimated cost is the increase in expected costs for the unprecedented vaccine candidates (i.e. the eight malaria vaccine candidates, five placental malaria vaccine candidates and two leishmaniasis vaccine candidates). Results for the vaccine candidates for Nipah virus, Zika virus, and shigellosis, ETEC were not affected by the change in these parameters.

The costs that we estimated using the P2I tool are likely to be an underestimate of the true costs. Vaccine development is a reiterative process, meaning that many steps, such as clinical trial phases, will be conducted several times. This process is in contrast to the rationale of the P2I tool, which assumes a straightforward development of product candidates without the reiteration of any particular development steps. For example, very often several phase I trials for a particular antigen are conducted, i.e. multiple trials in which different formulations, different routes of administration or different technology platforms for antigen presentation are being tested and compared. Also, very often several phase I clinical trials are conducted in which a vaccine´s safety is assessed in various age groups in an age-deescalating manner (i.e. the vaccine is tested consecutively in volunteer groups with decreasing age). It is therefore rather unlikely that any vaccine candidate would immediately advance to a phase II clinical trial after the conduct of a single phase I clinical trial only. Consequently, the total costs for the development of the different vaccine candidates in EVI´s portfolio are likely to be significantly higher than those estimated by the P2I tool. In addition, as several phase I or phase II clinical trials are likely to be conducted for the same vaccine (as explained above), due to such “reiterated” phases the overall timelines to reach the market are expected to be longer.

With regards to the estimated future launches, for all 18 candidates under development, the P2I model estimates that there would be 0.69 expected launches across all six diseases combined. In the re-run of the model using EVI‘s own internal parameters, thanks to higher success rates at EVI as compared to the P2I tool´s predefined parameters, the launch probability increased for malaria, placental malaria, and leishmaniasis vaccines (from 0.098 to 0.11, from 0.05 to 0.07, and from 0.024 to 0.03, respectively). Directly related to this increased launch probability, the total estimated costs for moving these vaccine candidates through the pipeline increased accordingly. This difference between the estimated future product launches emphasizes that, in order to increase the chances of ultimate success with this kind of product development, it is important to make the product development process technically as efficient as possible, i.e. reducing the attrition rates as much as possible. If attrition rates during product development cannot be reduced, the only other chance of achieving ultimate product launches is to boost the overall number of product candidates in the pipeline, obviously in the end resulting in higher total costs linked to the launch of a single product due to the high costs linked to failed product candidates.

However, rather than looking at the isolated results on likely launches from the analysis of a single organization’s portfolio, as has been done in this particular study, more meaningful results will be obtained from such simulations using the P2I tool by conducting a much wider portfolio analysis in which the launch estimation results of the *entire* global vaccine candidate portfolio are estimated in an integrated, combined manner. Only this kind of “full global portfolio” study can provide a reliable prediction of the product success rates on a global level for the next few decades.

The P2I tool has a number of other limitations, which were described in detail by Young *et al*. ^2^ We highlight six specific limitations. First, as a static, deterministic model, it does not take into account possible improvements in product development techniques over time (e.g. R&D efficiencies that reduce costs). Similarly, as a static model, it does not take into account the possibility that candidates may sometimes have to go “backwards” to an earlier phase. For example, once a candidate enters into phase I there may be bottlenecks that require that the candidate return to a preclinical evaluation stage (e.g., if different formulations need to be re-evaluated or alternate adjuvants need to be tested).

Second, the model’s assumptions for costs, attrition rates, and cycle times for phase are based on product development data from across multiple diseases (including non-communicable diseases—the model does not reflect possible differences in R&D parameters *between* different diseases). Although the assumptions were based on a very large number of data points (from 25,000 development candidates) and validated with experts, it is unclear how many of these data points came from vaccine development for neglected and emerging infectious diseases. Thus there is some uncertainty as to how accurate the assumptions are for the costs, attrition rates, and cycle times per phase for the three vaccine archetypes used in our study.

Third, the model does not include all phases of development (e.g. it excludes drug discovery, basic research, and regulatory review). The exclusion of phase IV studies, also known as post-marketing surveillance, is a major limitation—determining long-term safety and effectiveness is critical, yet it can be a lengthy, expensive process. We acknowledge that using the P2I tool, which only includes advanced pre-clinical to phase III, will always lead to an under-estimate of the total costs, since the costs of early pre-clinical research and post-phase III research can both be substantial. For example, based on data from a sample of 106 NCEs, DiMasi *et al.* ^5^ estimate that developing an NCE to the point of marketing approval costs $2.6 billion; this includes $1.2 billion in “time costs” (the expected returns that private investors forgo while a drug is in development) ^5^. Of this $2.6 billion, $1.1 billion is in pre-clinical development costs and $1.5 billion in clinical development costs. Previous research has suggested that the cost of regulatory approval stage may represent up to 5.7% of the total R&D cost ^6^.

By only including advanced pre-clinical to phase III, the model also provides no insight into the costs and complexity of the array of activities that need to happen after phase III for a new product to have a public health impact. The phase after phase III is often considered as a “valley of death” for product development—a product may pass successfully through phase III but then there may be no concerted, strategic plan for large-scale manufacturing or scale-up. Demand forecasting, developing a long-term business case, understanding the public health value of new products, and analysing the delivery system and scale-up approach are all critical components in determining the ultimate public health utility of a new health technology. We have previously noted that the P2I model “is “agnostic” when it comes to the public health value of the estimated launches—it cannot judge their clinical utility” ^2^.

Fourth, accurate classification of candidates into their archetypes can be challenging. As we previously noted, “the P2I v.2 model requires users to classify every candidate into an archetype, but categorizing candidates based on the archetype definitions was challenging—especially determining a candidate’s complexity. It will be helpful for future iterations of the model to include more fine-grained, detailed descriptions” ^2^.

Fifth, the model assumes that the costs, attrition rates, and cycle times per phase for vaccine development would be the same regardless of the setting where the study is done. Yet in reality, it is likely that these parameters would be different if a study were conducted in a high-, middle-, or low-income country. It would be helpful for future iterations of the P2I tool to incorporate these differences across study settings.

Sixth, the model also assumes that the costs of vaccine development per phase do not vary and are predictable. Yet there can be substantial variation and unpredictability in items such as the cost of manufacturing the product candidates and adjuvants, or the maintenance and quality control of clinical trial sites.

In this study, several adaptations to the P2I tool initially were considered with the aim of improving the tool´s usefulness and reliability. First, we considered making adaptations of the assumptions for success rates, costs and cycle times based on EVI’s longstanding experience with conducting studies in resource-limited, low-income settings. Second, we considered making adaptations for the same parameters based on (a) the fast-track clinical development strategy often used by EVI (consisting of a strategy in which the first-in-human evaluation involves a staggered multi-centre phase Ia/b clinical trial resulting in shorter timelines ^7, 8^), and (b) the inclusion of accelerated clinical testing based on controlled human infection models available, for example, for malaria ^9, 10^. Third, in the original proposal we considered including adaptations of the assumptions for costs and cycle times for vaccines that might be eligible for accelerated approval by regulatory authorities, for example the “Expedited Programs for Serious Conditions––Drugs and Biologics” from the US Food and Drug Administration ^11^.

In the end, we were able to do one re-run of the P2I model using EVI’s own parameters for success rates and cycle times for phase I clinical trials for unprecedented vaccines (70% and 17.4 months, respectively, compared to 50% and 24 months defined in the original P2I tool for unprecedented vaccines). However, although to date EVI has been involved in the conduct of over 30 clinical trials, data from only a limited number of studies could be used in the analyses conducted. When it came to estimating clinical trial costs, for example, for several trials it was not possible to extract the specific costs for preclinical or clinical trial activities out of the overall study costs.

Concerning the proposed modification of P2I parameters based on accelerated clinical testing using controlled human challenge models and on accelerated approval by regulators, we realized that although these two issues are likely to speed up vaccine development, currently there is not enough evidence/data based on which the P2I parameters could be adapted and analyses be run to assess their impact.

In conclusion, we found that despite the limitations discussed above, the P2I tool was flexible and adaptable enough to be used to study EVI’s portfolio. We believe that the P2I model represents a useful tool to analyze the portfolio of global health products under development. Findings from the analysis of the overall EVI vaccine portfolio using the P2I tool will be taken into consideration in the next revision of the EVI Strategic Plan, and estimations for individual vaccine candidates will inform decisions regarding whether or not to continue with the development of individual vaccine candidates once they reach major milestone or stage gating criteria. We expect that studies like ours will inform future updates of the model that will further increase its value for product developers, R&D funders, and decision makers.

## Data availability

All data underlying the results are available as part of the article and no additional source data are required.

The particular vaccine candidates included in this study have been anonymized for intellectual property reasons.

## Footnotes

^1 ^see: https://www.who.int/immunization/research/meetings_workshops/30_MenozziA_VIPS.pdf?ua=1

^2 ^ See: https://www.who.int/immunization/research/meetings_workshops/31_Giersing_TSE.pdf?ua=1

^3 ^See: https://www.who.int/tdr/publications/r_d_report/en/

## References

[1] Terry RF Yamey G Miyazaki-Krause R: Funding global health product R&D: the Portfolio-To-Impact Model (P2I), a new tool for modelling the impact of different research portfolios [version 2; peer review: 2 approved]. *Gates Open Res.* 2018;2:24. 10.12688/gatesopenres.12816.2 30234194 PMC6139376

[2] Young R Bekele T Gunn A: Developing new health technologies for neglected diseases: a pipeline portfolio review and cost model [version 1; peer review: 1 approved, 2 approved with reservations]. *Gates Open Res.* 2018;2:23. 10.12688/gatesopenres.12817.1 30234193 PMC6139384

[3] Mestre-Ferrandiz J Sussex J Towse A: The R&D Cost of a New Medicine. UK Office of Health Economics.2012 Reference Source

[4] See: http://www.euvaccine.eu/portfolio/vaccine-development.

[5] DiMasi JA Grabowski HG Hansen RW: Innovation in the pharmaceutical industry: New estimates of R&D costs. *J Health Econ.* 2016;47:20–33. 10.1016/j.jhealeco.2016.01.012 26928437

[6] Schuhmacher A Gassmann O Hinder M: A Review of the Pharmaceutical R&D Efficiency: Costs, Timelines, and Probabilities.In: *Value Creation in the Pharmaceutical Industry: The Critical Path to Innovation* Edited by Schuhmacher A, Hinder M, Gassmann O. Wiley-VCH Verlag GmbH & Co. KGaA.2016 10.1002/9783527693405.ch4

[7] Steiner-Monard V Kamaka K Karoui O: The Candidate Blood-stage Malaria Vaccine P27A Induces a Robust Humoral Response in a Fast Track to the Field Phase 1 Trial in Exposed and Nonexposed Volunteers. *Clin Infect Dis.* 2019;68(3):466–474. 10.1093/cid/ciy514 29945169

[8] Sirima SB Durier C Kara L: Safety and immunogenicity of a recombinant *Plasmodium falciparum* AMA1-DiCo malaria vaccine adjuvanted with GLA-SE or Alhydrogel® in European and African adults: A phase 1a/1b, randomized, double-blind multi-centre trial. *Vaccine.* 2017;35(45):6218–6227. 10.1016/j.vaccine.2017.09.027 28947345

[9] Roestenberg M Mordmüller B Ockenhouse C: The frontline of controlled human malaria infections: A report from the controlled human infection models Workshop in Leiden University Medical Centre 5 May 2016. *Vaccine.* 2017;35(51):7065–7069. 10.1016/j.vaccine.2017.10.093 29153778

[10] Hodgson SH Juma E Salim A: Lessons learnt from the first controlled human malaria infection study conducted in Nairobi, Kenya. *Malar J.* 2015;14:182. 10.1186/s12936-015-0671-x 25927522 PMC4416324

[11] Expedited Programs for Serious Conditions - Drugs and Biologics.US Food and Drug Administration,2017 Reference Source

