# Supplementary material for: Pipeline analysis of a vaccine candidate portfolio for diseases of poverty using the Portfolio-To-Impact modelling tool
Source: F1000Res. 2020 Feb 4;8:1066. Originally published 2019 Jul 11. [Version 2] doi: 10.12688/f1000research.19810.2 (PMC7043114; doi:10.12688/f1000research.19810.2)
Supplement: Peer review history

# Peer review: Reviewer response for version 2

**DOI:** 10.5256/f1000research.24213.r59536

**Published:** 2020-05-13

**Authors:** Gerald H. Voss

**Affiliations:** 1 TuBerculosis Vaccine Initiative (TBVI), Lelystad, The Netherlands

I would like to thank the authors for the useful clarifications.

Is the work clearly and accurately presented and does it cite the current literature?

Partly

If applicable, is the statistical analysis and its interpretation appropriate?

Not applicable

Are all the source data underlying the results available to ensure full reproducibility?

Yes

Is the study design appropriate and is the work technically sound?

Partly

Are the conclusions drawn adequately supported by the results?

Yes

Are sufficient details of methods and analysis provided to allow replication by others?

Yes

Reviewer Expertise:

Vaccine development, PRDs, global health

I confirm that I have read this submission and believe that I have an appropriate level of expertise to confirm that it is of an acceptable scientific standard.

# Peer review: Reviewer response for version 2

**DOI:** 10.5256/f1000research.24213.r59535

**Published:** 2020-02-24

**Authors:** Maria Elena Bottazzi

**Affiliations:** 1 Texas Children's Hospital Center for Vaccine Development, National School of Tropical Medicine, Baylor College of Medicine, Houston, TX, USA

Thanks to the authors for improving the manuscript and responding to the reviewer queries.

Is the work clearly and accurately presented and does it cite the current literature?

Partly

If applicable, is the statistical analysis and its interpretation appropriate?

Not applicable

Are all the source data underlying the results available to ensure full reproducibility?

Partly

Is the study design appropriate and is the work technically sound?

Yes

Are the conclusions drawn adequately supported by the results?

Partly

Are sufficient details of methods and analysis provided to allow replication by others?

Partly

Reviewer Expertise:

Neglected tropical diseases, vaccine development, product development, global health

I confirm that I have read this submission and believe that I have an appropriate level of expertise to confirm that it is of an acceptable scientific standard.

# Peer review: Reviewer response for version 2

**DOI:** 10.5256/f1000research.24213.r59534

**Published:** 2020-02-11

**Authors:** Jean-Louis Excler

**Affiliations:** 1 New Initiatives, International Vaccine Initiative, Seoul, South Korea

Thank you to have addressed most of my comments in the current version 2.

 Regarding clinical trial duration, there may still be a misunderstanding. I was not referring to Phase IV but to Phase II (prior to Phase III). Your manuscript refers to the duration from first enrolled to last visit. It does not include the time of analysis of basic safety data and of immunogenicity data, the latter being sometimes long for complex vaccines (e.g., HIV vaccines). At best, the immuno analysis may take 6-12 mo, cleaning of the data and statistical analysis may well take another 6-12mo. This is not taken into account in your duration.

 Regarding my last comment on CEPI and cost estimates, please refer to the article of Gouglas D, et al. Lancet Glob Health 2018; 6: e1386–96 ^1^.

Is the work clearly and accurately presented and does it cite the current literature?

Partly

If applicable, is the statistical analysis and its interpretation appropriate?

Not applicable

Are all the source data underlying the results available to ensure full reproducibility?

No

Is the study design appropriate and is the work technically sound?

Yes

Are the conclusions drawn adequately supported by the results?

Partly

Are sufficient details of methods and analysis provided to allow replication by others?

Partly

Reviewer Expertise:

Vaccine Development, Global Health

I confirm that I have read this submission and believe that I have an appropriate level of expertise to confirm that it is of an acceptable scientific standard.

## References

[1] : Estimating the cost of vaccine development against epidemic infectious diseases: a cost minimisation study. *The Lancet Global Health*.2018;6(12) : 10.1016/S2214-109X(18)30346-2 e1386-e1396 10.1016/S2214-109X(18)30346-2 PMC716481130342925

# Peer review: Reviewer response for version 1

**DOI:** 10.5256/f1000research.21731.r53116

**Published:** 2019-09-30

**Authors:** Maria Elena Bottazzi

**Affiliations:** 1 Texas Children's Hospital Center for Vaccine Development, National School of Tropical Medicine, Baylor College of Medicine, Houston, TX, USA

This manuscript attempts to describe the applicability and adaptability (with assumption modifications) of a recently developed computer (Excel)-based modelling tool called Portfolio-to-Impact (P21 - version 2) that has as objective to estimate the minimum funding needs (costs) to move a portfolio of target candidates (drugs or vaccines) from late stage preclinical up to phase 3 trials and estimate the number of targets with the potential to reach the launch stage. Of note, the authors mention that prior publications describe the P21 tools in detail for both Versions 1 and 2 and that a prior publication had already analyzed the application of the P21 tool using a broad portfolio of 538 PRND candidates.

 For this manuscript the authors use as a case study a selection of targets from the vaccine portfolio of the European Vaccine Initiative (EVI). They focus on 18 vaccine candidates (3 in preclinical, 11 in phase 1 and 4 in phase 2) and with three different categories: 15 as unprecedented vaccines (13 malaria and 2 leishmania), 2 as simple vaccines (Zika and nipah) and 1 as a complex vaccine (shigella/ETEC). As methodology, the authors compare the outputs obtained after using the original P21V2 tool assumptions (cycle time, cost and attrition rate) with a modified set of EVI assumptions.

 As a reviewer, I believe the paper has some potential usability and interest, especially for other PRND vaccine developers and PDPs and those with small portfolios. There are several major observations, however that require attention and/or clarification to strengthen the paper before it is to be indexed:

1. Even though the paper highlights that the tool was developed specifically for PRNDs, it lacks more detailed information, discussion and evaluation of the bottlenecks or considerations especially when the development is being done outside HICs and the likely the challenges that would be applicable when the vaccines could be developed partly in and with LMICs organizations. The examples using the EVI portfolio still leaves unclear where the different phases are done. What if preclinical is in Europe but the clinical in Africa, would it be different the cost or probability? 
2. The model and paper don’t seem to take into consideration nor discuss the variable and unpredictable costs during vaccine development. For example, cost of goods, manufacture, proprietary components such as adjuvants, stability, regulatory, quality control costs, all needed to maintain and even replenish the clinical lots during the transition into the advanced clinical stages. 
3. It also makes no mention on the complexity of the costs, time and probability needed to continuously mature the production processes and its QC testing and reach suitability for use throughout the different clinical stages and pre-launch or post-launch. For instance, when measuring the probability and time to launch, does this take into consideration where and who would be the large-scale manufacturer? Would there also be the need for manufacturing infrastructure?
4. Even though the manuscript speaks about using “time to launch” and “probability to launch”, this is very vague and offers no value to the reader since there is a big valley after phase 3 trials that is not discussed. The public health value proposition, business case and demand forecasting for PRNDs is a very complex system that require the involvement of multiple WHO offices and committees, GAVI and others. This should be better elaborate in the manuscript. 
5. The manuscript lacks to elaborate on the reality that even once a product enters into Phase 1 there are bottlenecks, which may require that a product returns to a preclinical evaluation stage, for example if different formulations need to be re-evaluated or alternate adjuvants need to be tested. It also is not clear about costs and time when the clinical development is in resource-poor areas where regulatory hurdles may hamper the timelines.
6. Step 2 method description and table 3: if in the first run, the authors are using “exactly the same assumptions as P21V2 model”, it is unclear why there is also reference to two additional set of assumptions: the RAP and the BMGF assumptions. Are these readily available? If not, this would make the applicability of P21V2 obsolete especially for other PDPs and PRND vaccine developers with smaller or non-gates supported portfolios.
7. Step 3 method description and table 4: the authors mention that the modified EVI assumptions may be unreliable because they are based only on 2-3 data points and decided to make a pragmatic decision to use only those with 10 data points. This in itself is flawed. If the objective of the authors is to showcase how the tool assumptions or a modification of tool assumptions could support vaccine producers in their forecasting exercise, the model should be based on close to reality, especially since most PRND vaccine portfolios do not contain large number of vaccine candidates or data points. The authors should address and comment on this especially since it seems the probability rates are too generous in the EVI assumptions.
8. The disease focus of the selected portfolio is quite varied and for some (ie malaria) with multiple targets versus for some only one target. A more detail discussion on the applicability of the tool for the evaluation of 1-2 targets versus >2 targets would be very useful.

Is the work clearly and accurately presented and does it cite the current literature?

Partly

If applicable, is the statistical analysis and its interpretation appropriate?

Not applicable

Are all the source data underlying the results available to ensure full reproducibility?

Partly

Is the study design appropriate and is the work technically sound?

Yes

Are the conclusions drawn adequately supported by the results?

Partly

Are sufficient details of methods and analysis provided to allow replication by others?

Partly

Reviewer Expertise:

Neglected tropical diseases, vaccine development, product development, global health

I confirm that I have read this submission and believe that I have an appropriate level of expertise to state that I do not consider it to be of an acceptable scientific standard, for reasons outlined above.

# Response

**Published:** 2020-01-03

**Authors:** Stefan Jungbluth

**Affiliations:** European Vaccine Initiative (EVI), Germany

**Comment:**

 Even though the paper highlights that the tool was developed specifically for PRNDs, it lacks more detailed information, discussion and evaluation of the bottlenecks or considerations especially when the development is being done outside HICs and the likely the challenges that would be applicable when the vaccines could be developed partly in and with LMICs organizations. The examples using the EVI portfolio still leaves unclear where the different phases are done. What if preclinical is in Europe but the clinical in Africa, would it be different the cost or probability? 

**Response from authors:**

 This is an excellent point.  The model is “agnostic” as to where the development is being done, which is a limitation. In the revision, we now acknowledge this limitation in the Discussion section (in the discussion of limitations). We write:

 “Fifth, the model assumes that the costs, attrition rates, and cycle times per phase for vaccine development would be the same regardless of the setting where the study is done. Yet in reality, it is likely that these parameters would be different if a study was conducted in a high-, middle-, or low-income country. It would be helpful for future iterations of the P2I tool to incorporate these differences across study settings.”

**Comment:**

 The model and paper don’t seem to take into consideration nor discuss the variable and unpredictable costs during vaccine development. For example, cost of goods, manufacture, proprietary components such as adjuvants, stability, regulatory, quality control costs, all needed to maintain and even replenish the clinical lots during the transition into the advanced clinical stages.   It also makes no mention on the complexity of the costs, time and probability needed to continuously mature the production processes and its QC testing and reach suitability for use throughout the different clinical stages and pre-launch or post-launch. For instance, when measuring the probability and time to launch, does this take into consideration where and who would be the large-scale manufacturer? Would there also be the need for manufacturing infrastructure?

**Response from authors:**

 This is another excellent point.  We now discuss this in the limitations section of the discussion.  We write:

 “Sixth, the model also assumes that the costs of vaccine development per phase do not vary and are predictable. Yet there can be substantial variation and unpredictability in items such as the cost of manufacturing the product candidates and adjuvants, or the maintenance and quality control of clinical trial sites.”

 As discussed in detail in the revision, there are many costs that are excluded from the P2I model, which is indeed a limitation. The model only includes advanced clinical to phase III (it does not include post-phase III large-scale manufacturing, regulatory approval, marketing, etc.). We state:

 “Third, the model does not include all phases of development (e.g. it excludes discovery, basic research, and regulatory review). The exclusion of phase IV studies, also known as post-marketing surveillance, is a major limitation—determining long-term safety and effectiveness is critical, yet it can be a lengthy, expensive process. We acknowledge that using the P2I tool, which only includes advanced pre-clinical to phase III, will always lead to an under-estimate of the total costs, since the costs of early pre-clinical research and post-phase III research can both be substantial. For example, based on data from a sample of 106 NCEs, DiMasi *et al* estimate that developing an NCE to the point of marketing approval costs $2.6 billion to, which includes $1.2 billion in “time costs” (the expected returns that private investors forgo while a drug is in development). ^10^ Of this $2.6 billion, $1.1 billion is in pre-clinical development costs and $1.5 billion in clinical development costs.  Previous research has suggested that the cost of regulatory approval stage may represent up to 5.7% of the total R&D cost. ^11^”

**Comment:**

 Even though the manuscript speaks about using “time to launch” and “probability to launch”, this is very vague and offers no value to the reader since there is a big valley after phase 3 trials that is not discussed. The public health value proposition, business case and demand forecasting for PRNDs is a very complex system that require the involvement of multiple WHO offices and committees, GAVI and others. This should be better elaborate in the manuscript. 

**Response from authors:**

 In the revision, we now make it much clearer what we mean by launch (i.e. a candidate makes it through phase III and is ready for post-phase III steps, such as regulatory review). We also now, in the discussion, explore the valley after phase III. In the revision, we now state:

 “By only including advanced pre-clinical to phase III, the model also provides no insight into the costs and complexity of the array of activities that need to happen after phase III for a new product to have a public health impact.  The phase after phase III is often considered as a “valley of death” for product development—a product may pass successfully through phase III but then there is no concerted, strategic plan for large-scale manufacturing or scale-up. Demand forecasting, developing a long-term business case, understanding the public health value of new products, and analysing the delivery system and scale-up approach are all critical components in determining the ultimate public health utility of a new health technology. We have previously noted that the P2I model “is “agnostic” when it comes to the public health value of the estimated launches—it cannot judge their clinical utility.” ^2”^

**Comment:**

 The manuscript lacks to elaborate on the reality that even once a product enters into Phase 1 there are bottlenecks, which may require that a product returns to a preclinical evaluation stage, for example if different formulations need to be re-evaluated or alternate adjuvants need to be tested. It also is not clear about costs and time when the clinical development is in resource-poor areas where regulatory hurdles may hamper the timelines

**Response from authors:**

 This is an excellent point, which we now address in the revised discussion section:

 “The P2I tool has a number of other limitations, which were described in detail by Young *et al*. ^2^ We highlight X specific limitations. First, as a static, deterministic model, it does not take into account possible improvements in product development techniques over time (e.g. R&D efficiencies that reduce costs). Similarly, as a static model, it does not take into account the possibility that candidates may sometimes have to go “backwards” to an earlier phase. For example, once a candidate enters into Phase I there may be bottlenecks that require that the candidate return to a preclinical evaluation stage (e.g., if different formulations need to be re-evaluated or alternate adjuvants need to be tested).”

**Comment:**

 Step 2 method description and table 3: if in the first run, the authors are using “exactly the same assumptions as P21V2 model”, it is unclear why there is also reference to two additional set of assumptions: the RAP and the BMGF assumptions. Are these readily available? If not, this would make the applicability of P21V2 obsolete especially for other PDPs and PRND vaccine developers with smaller or non-gates supported portfolios.

**Response from authors:**

 We now discuss the RAP and the Gates Foundation as data sources for P2I.v2; see the revised Methods section.  The RAP is not public; the Gates Foundation data are shown in reference 2.

**Comment:**

 Authors mention that the modified EVI assumptions may be unreliable because they are based only on 2-3 data points and decided to make a pragmatic decision to use only those with 10 data points. This in itself is flawed etc

**Response from authors:**

 We agree that model should be based on real data, however we do not consider it as meaningful to modify the P2I tool assumptions if such alternative parameters only include 2-3 data points.

 A better way forward will be to combine these low numbers of alternative data points with additional data points coming from other users of the tool, thereby increasing the overall total number of data points available. Such a combined, increased number of data points can then be used as alternative parameters for the analysis of product portfolios

**Comment:**

 Probability rates are too generous in the EVI assumptions.

**Response from authors:**

 Probability rates included are the real success rates obtained at EVI. These high success rates are also not surprising, given that they relate to phase I clinical trials that test for vaccine safety where success rates usually are rather high.

**Comment:**

 Disease focus of the selected portfolio is quite varied and for some (ie malaria) with multiple targets versus for some only one target. A more detail discussion on the applicability of the tool for the evaluation of 1-2 targets versus >2 targets would be very useful.

**Response from authors:**

 Wording that had already been included in the article has been slightly modified for clarification. As mentioned in the article, especially for the estimation of expected product launches the P2I tool should be used for an overall analysis of global product candidate portfolio, compared to the analysis of individual organisation´s portfolio only. Such combined global studies will provide far more meaningful data.

# Peer review: Reviewer response for version 1

**DOI:** 10.5256/f1000research.21731.r51927

**Published:** 2019-08-19

**Authors:** Gerald H. Voss

**Affiliations:** 1 TuBerculosis Vaccine Initiative (TBVI), Lelystad, The Netherlands

This article describes the application of the P2I model to the vaccine development portfolio of EVI. The intent of the exercise is to provide a portfolio funding estimate from late preclinical through completion of phase III. The authors proceed in four steps by classifying their vaccine candidates into archetypes, running the standard P2I v2 model, re-running the model using modified assumptions based on EVI's own data, and conducting a sensitivity analysis for the model assumptions. The authors conclude that the P2I model, despite several limitations, is suitable and sufficiently flexible to study EVI's vaccine portfolio.

 There are a number of methodological questions that merit further consideration or clarification. 

 First, the notion of product launch is vague. The model calculates costs from late preclinical up to completion of phase III, so this approach seems to provide numbers for 'launch readiness' rather than product launch and should be clearly stated.

 Second, significant other costs for vaccine development do not seem to be included, for example process development, GMP lots and investment into manufacturing. Due to this limitation, the calculated funding needs provide only a partial view of the overall required investment.

 Third, clinical phase duration does not take into account the time to prepare for clinical trials, perform data analysis and generate a study report. Together with the author's comment that each clinical trial phase is likely to comprise several iterative studies, the time (and cost) assumptions appear very optimistic.

 Fourth, the classification into archetypes is somewhat arbitrary. One could argue that the RTS,S and M72 (out of scope of this exercise) vaccines provide precedents for malaria and TB, respectively, and should increase the phase II and III probability of success assumptions.

 Overall, this study seems to demonstrate that the usefulness of any model depends on the quality of the assumptions. Unfortunately, as the authors indicate, there is a scarcity of data in type II and III disease vaccine development, thereby rendering the generation of assumptions unreliable.

 In the reviewer's opinion, the article would benefit from answering two questions: how has the model helped EVI to prioritise its portfolio and how can the model be improved.

 Minor comments:

- Table 5 and figures 2 and 3 are redundant
- Table 6 and figure 4 are redundant. It would also help to clarify what 'cost' and 'cumulative annual' means. Is that 'cumulative cost'?
- Table 7 'of' needs to be deleted in the title

Is the work clearly and accurately presented and does it cite the current literature?

Partly

If applicable, is the statistical analysis and its interpretation appropriate?

Not applicable

Are all the source data underlying the results available to ensure full reproducibility?

Yes

Is the study design appropriate and is the work technically sound?

Partly

Are the conclusions drawn adequately supported by the results?

Yes

Are sufficient details of methods and analysis provided to allow replication by others?

Yes

Reviewer Expertise:

Vaccine development, PRDs, global health

I confirm that I have read this submission and believe that I have an appropriate level of expertise to confirm that it is of an acceptable scientific standard, however I have significant reservations, as outlined above.

# Response

**Published:** 2020-01-03

**Authors:** Stefan Jungbluth

**Affiliations:** European Vaccine Initiative (EVI), Germany

**Comment:**

 First, the notion of product launch is vague. The model calculates costs from late preclinical up to completion of phase III, so this approach seems to provide numbers for 'launch readiness' rather than product launch and should be clearly stated

**Response from authors:**

 Reviewer 1 also raised this point. We now explain this more clearly. We state: “in this paper, the term launch refers to a candidate making it through phase III and thus being ready for the next steps, e.g. regulatory and manufacturing steps.”

**Comment:**

 Second, significant other costs for vaccine development do not seem to be included, for example process development, GMP lots and investment into manufacturing. Due to this limitation, the calculated funding needs provide only a partial view of the overall required investment.

**Response from authors:**

 The reviewer is correct, and this is one of the limitations of the P2I model. We now state this limitation clearly in the methods, and discuss its implications in the Discussion section.  In the revised Methods, we now state: “The model includes only advanced preclinical to phase III, and thus the cost estimates are an under-estimate of the full costs of product development. In particular, the model excludes all costs related to basic research through lead optimization; chemistry, manufacturing, and controls; good manufacturing practice; manufacturing build up and scale-up costs; regulatory or registration fees (post-phase III); and all post-market commitments (e.g., phase IV pharmacovigilance studies).”

**Comment:**

 Third, clinical phase duration does not take into account the time to prepare for clinical trials, perform data analysis and generate a study report. Together with the author's comment that each clinical trial phase is likely to comprise several iterative studies, the time (and cost) assumptions appear very optimistic.

**Response from authors:**

 In the revised paper, we now explain exactly how these assumptions were generated and validated (and also point readers to the two previous research articles that explain these assumptions in greater detail).

**Comment:**

 Fourth, the classification into archetypes is somewhat arbitrary. One could argue that the RTS,S and M72 (out of scope of this exercise) vaccines provide precedents for malaria and TB, respectively, and should increase the phase II and III probability of success assumptions.

**Response from authors:**

 We respectfully disagree that classification is arbitrary, and we now refer readers to our previous two studies showing more details on the classification process. But we certainly do agree that classification is an imperfect science, and we now discuss this limitation in the revised Discussion section.

**Comment:**

 Overall, this study seems to demonstrate that the usefulness of any model depends on the quality of the assumptions. Unfortunately, as the authors indicate, there is a scarcity of data in type II and III disease vaccine development, thereby rendering the generation of assumptions unreliable.

**Response from authors:**

 We agree that all models are dependent on the quality of the model assumptions. We now explain in greater detail how these were derived and validated. We respectfully disagree that the assumptions are unreliable; indeed, we know of no author assumptions that are more reliable than those in the P2I model. We now state, in the revision: “As described in detail in reference 1, assumptions on development costs at each phase of product development for the 11 archetypes included in the P2I.v1 model were initially based on an analysis of clinical trial costs from Parexel’s R&D cost sourcebook. The assumptions on attrition rates and cycle times at each phase were initially based on the historical attrition rates and cycle times of more than 25,000 development candidates. All of these assumptions were further refined and validated based on academic literature, industry publications and databases, and 133 stakeholder interviews with a wide variety of product development partnerships (PDPs), pharmaceutical companies, and major funders of global health R&D. As described in detail in reference 2, additional sources of assumptions for the new archetypes in P2I.v2 were derived from the McKinsey Risk-Adjusted Portfolio (RAP) Model and from clinical trial data shared with us by the Bill & Melinda Gates Foundation.”

 In the discussion section, when we discuss limitations, in the revision we now write: “the model’s assumptions for costs, attrition rates, and cycle times for phase are based on product development data from across multiple diseases (including non-communicable diseases—the model does not reflect possible differences in R&D parameters *between* different diseases). Although the assumptions were based on a very large number of data points (from 25,000 development candidates) and validated with experts, it is unclear how many of these data points came from vaccine development for neglected and emerging infectious diseases. Thus there is some uncertainty as to how accurate the assumptions are for the costs, attrition rates, and cycle times per phase for the three vaccine archetypes used in our study.”

**Comment:**

 How has the model helped EVI to prioritise its portfolio?

**Response from authors:**

 Corresponding wording has been included in the article. At this particular moment, the analysis with the P2I tool is too recent and has not yet been taken into account in any particular decision making concerning the vaccine portfolio.

**Comment:**

 How can the model be improved?

**Response authors:**

 The Discussion part of the article already contained a lengthy section where weaknesses and potential improvements to the tool are being discussed

 Other minor suggestions (spelling etc.) made by the reviewer have been included in the revised article.  At the reviewer’s suggestion, we have removed figures 2-4. We have also taken out the word “cumulative” throughout the paper, as it was confusing.

# Peer review: Reviewer response for version 1

**DOI:** 10.5256/f1000research.21731.r52206

**Published:** 2019-08-13

**Authors:** Jean-Louis Excler

**Affiliations:** 1 New Initiatives, International Vaccine Initiative, Seoul, South Korea

The authors use the Portfolio-To-Impact (P2I) version model to estimate funding needs to move candidate vaccines to potential launch. P2I is a cost modeling tool. 

 It is unclear to a reader unfamiliar with this tool who are the target stakeholders of the outcome of the analysis: manufacturers, non-for-profit developers, donors, public health policy makers? Although the authors provide references, this should be summarized and clarified. 

 It is also unclear how this tool articulates for a given vaccine with the development of the Business Case ****component of the Full Public Health Value Proposition and the Total Systems Effectiveness (TSE) and the Vaccine Innovation Prioritization Strategy (VIPS) developed by WHO. What is the added value of the P2I model compared to the other models referred in Table 3? This should also be clarified. 

 It might also be useful to remind how EVI prioritized their vaccine candidate portfolio (no reference cited) and why they selected some of them for this P2I analysis. 

 The clinical trial duration as defined on page 6 may be misleading. The added duration of safety and immunogenicity analysis (the latter may be long) is not taken into account while these are key data driving the next phases of development.

 The term "launch" may deserve some clarification. Are the authors talking about registration for launch on the public market in LMICs, private market in LMICs, or per Gavi scheme for eligible countries? What about MICs which are no longer Gave-eligible and whose list is increasing? It is unclear whether the model considers vaccine manufacturing by traditional big western pharmas or developing country vaccine manufacturers (DCVM) and consider bringing the vaccine to WHO prequalification. This latter point is of importance as often this may require additional clinical studies adding to the overall development costs and duration before launch. 

 It is difficult to understand Tables 6-7 (Title of Table 7: delete 'of' based...) and Figures 3-4 of 'expected launches'. What do the data presented mean? For example, in text '0.69'. Is it a probability?

 Similarly Tables 8 and 9 would need ample explanation about what is considered in these tables. 

 Coming back to the estimated cost of vaccine development to launch, it is difficult to weigh the value of the findings compared to real life costs. For example, the cost of RTS,S malaria vaccine developed by GSK is estimated to be close to $500M. For Nipah and Zika, what is the development cost estimated by CEPI? How does it compare to EVI estimates? What the P2I model ever tested a posteriori for vaccines already developed and for which the development cost is know in order to measure the accuracy of the model prediction?

Is the work clearly and accurately presented and does it cite the current literature?

Partly

If applicable, is the statistical analysis and its interpretation appropriate?

Not applicable

Are all the source data underlying the results available to ensure full reproducibility?

No

Is the study design appropriate and is the work technically sound?

Yes

Are the conclusions drawn adequately supported by the results?

Partly

Are sufficient details of methods and analysis provided to allow replication by others?

Partly

Reviewer Expertise:

Vaccine Development, Global Health

I confirm that I have read this submission and believe that I have an appropriate level of expertise to state that I do not consider it to be of an acceptable scientific standard, for reasons outlined above.

# Response

**Published:** 2020-01-03

**Authors:** Stefan Jungbluth

**Affiliations:** European Vaccine Initiative (EVI), Germany

**Comment:**

 It is unclear to a reader unfamiliar with this tool who are the target stakeholders of the outcome of the analysis: manufacturers, non-for-profit developers, donors, public health policy makers? Although the authors provide references, this should be summarized and clarified

**Response from authors:**

 We have added a sentence in the introduction to explain this. We now state: “As a financial forecasting tool that estimates the funding needs for pharmaceutical product development, the tool and its outputs are of value to funders of product development, product development partnerships, and other stakeholders involved in research and development (R&D) policy. Terry and colleagues, the developers of the P2I model, note that “its real utility lies in its predictive value for modelling the impact of different funding strategies at the portfolio level.” ^1^

**Comment:**

 It is also unclear how this tool articulates for a given vaccine with the development of the Business Case component of the Full Public Health Value Proposition and the Total Systems Effectiveness (TSE) and the Vaccine Innovation Prioritization Strategy (VIPS) developed by WHO.

**Response from authors:**

 In the introduction, we have now added: “We used the P2I tool because, to the best of our knowledge, it is the first publicly available comprehensive portfolio model that includes data on cost, success rate, and cycle time per phase for various product types based on data from a very large number of previous product development candidates (over 25,000) ^1^. The P2I tool is thus complementary to other available tools that can help guide prioritization in vaccine development, such as the multi-stakeholder Vaccine Innovation Prioritization Strategy and Total Systems Effectiveness Framework.”

**Comment:**

 What is the added value of the P2I model compared to the other models referred in Table 3? This should also be clarified. 

**Response from authors:**

 Table 3 does not show other models. It shows the P2I model’s underlying assumptions on cost, cycle time, and success rate per development phase for three different categories of vaccines (simple, complex, and unprecedented). It also shows the source of data for these assumptions. In the text, we state: “In this first run of the model, we used exactly the same assumptions on cycle time, cost, and attrition rate per phase as in the P2I.v2 model ^2^ . These assumptions are shown in Table 3. The assumptions were derived from three sources: the P2I model (shown in orange in Table 3), the McKinsey Risk-Adjusted Portfolio (RAP) Model (shown in yellow), and the Bill & Melinda Gates Foundation (shown in blue) ^2^ .”

**Comment:**

 It might also be useful to remind how EVI prioritized their vaccine candidate portfolio (no reference cited) and why they selected some of them for this P2I analysis.

**Response from authors:**

 New wording describing the vaccine candidate selection and priorisation process, including a reference, have been included in the discussion. Reasons re the selection of vaccine candidates that were included in this study were already provided in the original article.

**Comment:**

 The clinical trial duration as defined on page 6 may be misleading. The added duration of safety and immunogenicity analysis (the latter may be long) is not taken into account while these are key data driving the next phases of development.

**Response from authors:**

 This is a valid point. The model only includes advanced clinical to phase III (it does not include phase IV (examining for safety and side effects). We now discuss this limitation in greater detail in the discussion. In the discussion section, we have added: “The exclusion of phase IV studies, also known as post-marketing surveillance, is a major limitation—determining long-term safety and effectiveness is critical, yet it can be a lengthy, expensive process.”

**Comment:**

 The term "launch" may deserve some clarification. Are the authors talking about registration for launch on the public market in LMICs, private market in LMICs, or per Gavi scheme for eligible countries? What about MICs which are no longer Gavi-eligible and whose list is increasing? It is unclear whether the model considers vaccine manufacturing by traditional big western pharmas or developing country vaccine manufacturers (DCVM) and consider bringing the vaccine to WHO prequalification. This latter point is of importance as often this may require additional clinical studies adding to the overall development costs and duration before launch. 

**Response from authors:**

 This is an excellent point (reviewer 2 also raised this point); we have now clarified this in more detail. Several reviewers asked for more details about what was included and what was excluded from the P2I model. This level of detail is in the original paper that describes the P2I model development ( https://gatesopenresearch.org/articles/2-24/v2), but since the reviewers has asked to see the details, we have now included these in our revised paper.

 We use the word “launch” here to refer to a candidate making it through Phase III, i.e., it is “launch ready,” which we now state in the revised paper. We have added a figure to show which phases are included in the P2I model. The P2I model excludes all costs related to basic research through lead optimization; chemistry, manufacturing, and controls; good manufacturing practice; manufacturing build up and scale-up costs; regulatory or registration fees (post-phase III); and all post-market commitments (e.g., phase IV pharmacovigilance studies).

**Comment:**

 It is difficult to understand Tables 6-7 (Title of Table 7: delete 'of' based...) and Figures 3-4 of 'expected launches'. What do the data presented mean? For example, in text '0.69'. Is it a probability? Similarly Tables 8 and 9 would need ample explanation about what is considered in these tables. 

**Response from authors:**

 We have fixed the typo, we have clarified that these are indeed launch probabilities, and we have now added much greater explanation of the findings shown in Tables 6-9 and figures 3-4.

**Comment:**

 For Nipah and Zika, what is the development cost estimated by CEPI? How does it compare to EVI estimates?

**Response from authors:**

 As far as we know, no estimated costs for the total or different steps are available from CEPI for the development of Nipah and Zika vaccines. Also, such costs would vary significantly, depending on the particular vaccine technology employed for vaccine development (eg protein-based vs. DNA/RNA based vs. viral vector etc), as well as on many other factors.

 Data one can obtain from the CEPI web page are the total maximum funding amounts awarded by CEPI for the different Nipah and Zika vaccines that currently are in the CEPI portfolio. However, we do not consider these figures as meaningful comparators for our estimations as the details of the funded projects (eg vaccine technology, other specific activities included in different projects etc) are not known to us and it is therefore unclear how the vaccines funded by CEPI compare to the one in EVI´s portfolio (NB: The Nipah vaccine candidate in EVI´s portfolio is funded by CEPI but vaccine development is only funded down to phase II clinical trial).

**Comment:**

 What the P2I model ever tested *a posteriori* for vaccines already developed and for which the development cost is known in order to measure the accuracy of the model prediction?

**Response from authors:**

 No vaccines that have finalised their development are available at EVI for such an *a posteriori* analysis, so for the time being this issue cannot be addressed.
